# Performance comparison of four commercial human whole-exome capture platforms

**DOI:** 10.1038/srep12742

**Published:** 2015-08-03

**Authors:** Daichi Shigemizu, Yukihide Momozawa, Testuo Abe, Takashi Morizono, Keith A. Boroevich, Sadaaki Takata, Kyota Ashikawa, Michiaki Kubo, Tatsuhiko Tsunoda

**Affiliations:** 1 Laboratory for Medical Science Mathematics, RIKEN Center for Integrative Medical Sciences, Yokohama, Japan; 2 Laboratory for Genotyping Development, RIKEN Center for Integrative Medical Sciences, Yokohama, Japan

## Abstract

Whole exome sequencing (WXS) is widely used to identify causative genetic mutations of diseases. However, not only have several commercial human exome capture platforms been developed, but substantial updates have been released in the past few years. We report a performance comparison for the latest release of four commercial platforms, Roche/NimbleGen’s SeqCap EZ Human Exome Library v3.0, Illumina’s Nextera Rapid Capture Exome (v1.2), Agilent’s SureSelect XT Human All Exon v5 and Agilent’s SureSelect QXT, using the same DNA samples. Agilent XT showed the highest target enrichment efficiency and the best SNV and short indel detection sensitivity in coding regions with the least amount of sequencing. Agilent QXT had slightly inferior target enrichment than Agilent XT. Illumina, with additional sequencing, detected SNVs and short indels at the same quality as Agilent XT, and showed the best performance in coverage of medically interesting mutations. NimbleGen detected more SNVs and indels in untranslated regions than the others. We also found that the platforms, which enzymatically fragment the genomic DNA (gDNA), detected more homozygous SNVs than those using sonicated gDNA. We believe that our analysis will help investigators when selecting a suitable exome capture platform for their particular research.

The detection of variants in diseased individuals is one of the important milestones of many genetic studies, because these genetic variants/mutations play an important role in human diseases and in particular rare disorders^1^,^2^. Next-generation sequencing technologies^3^,^4^,^5^ have enabled the whole genome sequencing (WGS) of the many individuals. Although WGS can identify various kinds of genetic variations, including single nucleotide variation (SNV), insertion and deletion (indel), copy number variation and rearrangement, and provide a list of genetic variations from the entire genomic region, cost for sequencing and data analysis still remains a key consideration^1^,^6^,^7^. Therefore, whole exome sequencing (WXS), which captures and sequences only the coding exons of the genome, has been recently widely used as one alternative to WGS^8^, since WXS is more cost-effective than WGS and approximately 85% of the genetic mutations underlying human diseases can be found in coding regions or in intronic splice-sites^1^,^9^. In fact, the WXS contributes to approximately five times lower cost than WGS^8^ and has successfully identified causal mutations of multiple Mendelian diseases^2^,^9^ and tumors^6^,^10^,^11^,^12^.

Currently, there are four major exome enrichment platforms: Roche/NimbleGen’s SeqCap EZ Human Exome Library, Illumina’s Nextera Rapid Capture Exome, Agilent’s SureSelect XT Human All Exon and Agilent’s SureSelect QXT. These platforms use biotinylated DNA or RNA baits complementary to the target exome, which are hybridized to genomic fragment libraries. The main differences are target region selection, bait length, bait density, molecule used for capture, and genomic fragmentation method. Several performance comparison studies among these exome capture platforms have been reported^13^,^14^,^15^,^16^,^17^. However, substantial updates have been released for each of these platforms over the past few years.

Chilamakuri, C.S. *et al.* recently reported a performance comparison of four commercial exome platforms: Roche/NimbleGen’s SeqCap EZ Human Exome Library v3.0, Illumina’s Nextera Rapid Capture Exome, Illumina’s TruSeq Exome Enrichment kit, and Agilent’s SureSelect XT2 Human All Exon v4. Here, we further systematically analyzed the latest four exome enrichment platforms: Roche/NimbleGen’s SeqCap EZ Human Exome Library v3.0, Illumina’s Nextera Rapid Capture Exome (v1.2), Agilent’s SureSelect XT Human All Exon v5, and Agilent’s SureSelect QXT. We compared differences in target region design, target enrichment efficiency, GC bias, variant discovery and medically interesting mutation discovery among these platforms.

## Results

### Designed target regions with exome capture platform

**Figure 1:**
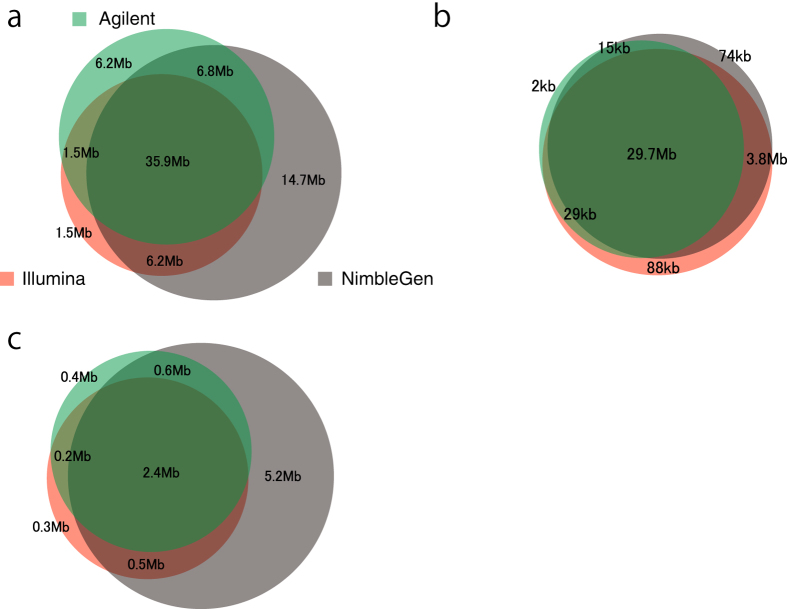
Venn diagram showing the overlap of targeted bases for all platforms. (**a**) Targeted genomic regions, (**b**) targeted coding regions and (**c**) targeted untranslated regions (UTR) in all platforms. The total targeted genomic regions were 63,564,965 bases for NimbleGen, 50,390,601 bases for Agilent, and 45,112,692 bases for Illumina.

Each commercial exome capture platform has different set of target regions, although Agilent XT and Agilent QXT use the same target regions. We compared the target regions of each platform based on the design documents obtained from the respective companies. Of the target regions, a large number of bases (35.9 Mb) were shared among all platforms. The NimbleGen platform possessed the most platform-specific target regions compared to the other platforms (NimbleGen-specific; 14.7 Mb, Agilent-specific; 6.2 Mb, Illumina-specific; 1.5 Mb, Fig. 1a). We categorized the target regions into coding regions and untranslated regions (UTRs) using NCBI’s reference sequence database (RefSeq) and separately compared the coverage among platforms. Most of the coding regions were represented on all platforms, which accounted for 29.7 Mb out of 33.5 Mb (Fig. 1b). Both the Illumina and NimbleGen platforms covered 3.8 Mb more coding regions than the Agilent platform (Fig. 1b). For UTRs, the NimbleGen platform has the greatest amount of platform-specific targeted bases (8.7 Mb), whereas the Illumina and Agilent platforms targeted 3.6 Mb and 3.4 Mb of UTRs, respectively (Fig. 1c).

### Sequencing coverage of on-target regions

To assess the sequencing coverage of on-target regions (regions including 100bp flanking regions from both ends of target regions) for each platform, we applied each target-enrichment platform to the same two individuals (NA18943 and NA18948) and sequenced them using the Illumina HiSeq2500 platform with paired-end reads of 161 bp. For each platform, 112–184 million (M) reads were obtained. Generally, 75 M reads (12 Gb) were randomly selected from each data set for comparison. When necessary, 25 M reads (4 Gb), 50 M reads (8 Gb), and 100 M reads (16 Gb) were also randomly selected from each data set.

**Figure 2:**
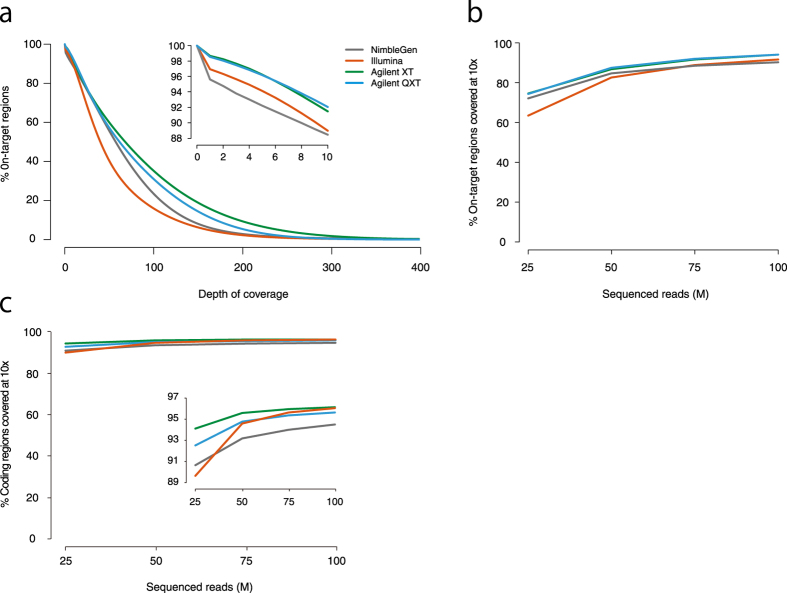
Coverage of target regions for each platform for NA18943. (**a**) The percent of total targeted bases covered with more than or equal to specified depths in the NA18943 sample. For target regions, 88.4% had at least ten times sequence coverage with NimbleGen, 88.9% with Illumina, 91.5% with Agilent XT, and 92.0% with Agilent QXT. The percent of on-target regions (**b**) and coding regions (**c**) covered with at least 10-fold read depth at increasing read counts. When 75 M reads were sequenced, 94.0% of coding regions had at least ten times coverage on average with NimbleGen, 95.7% with Illumina, 96.0% with Agilent XT and 95.4% with Agilent QXT.

For each platform, 96.4%–98.9% of the 75 M reads were mapped to the human reference genome, and 3.2%–14.9% were removed as duplicate PCR reads (Supplementary Table S1). In general, these values appear to be within the acceptable range: when a high level of PCR duplicates is over 30%, it is often dependent on the quality of the genomic DNA (gDNA). However, to be thorough we examined the variability of the PCR duplication rate. We resequenced the same samples with Illumina platform and calculated the number of PCR duplications. The PCR duplicate counts were different than previously sequenced data (3.99% and 9.23% for NA18943; 3.74% and 8.84% for NA18948, Supplementary Table S1), indicating that the level of PCR duplicates varies even for the same sample sequenced by the same platform. More than 88.0% of the on-target regions had at least ten times sequence coverage in all platforms (Fig. 2a for NA18943 and Supplementary Fig. S1a for NA18948).

Sequence coverage of coding regions is of most importance in WXS analysis. Therefore, we investigated the percentage of on-target regions (Fig. 2b for NA18943 and Supplementary Fig. S1b for NA18948) and coding regions included in the on-target regions (Fig. 2c for NA18943 and Supplementary Fig. S1c for NA18948) with at least ten times coverage from a randomly selected set of 25 M reads (4 Gb), 50 M reads (8 Gb), 75 M reads (12 Gb) and 100 M reads (16 Gb) from each data set. The Agilent XT platform achieved the highest sequence coverage in the coding regions. The Illumina platform achieved the same sequence coverage as the Agilent XT in coding regions when additional sequence was obtained. When 75 M reads were sequenced, 94.0% of all transcripts in NA18943 had at least ten times coverage on average with NimbleGen, 95.7% with Illumina, 96.0% with Agilent XT and 95.4% with Agilent QXT (Fig. 2c for NA18943 and Supplementary Fig. S1c for NA18948).

### Target enrichment efficiency

**Figure 3:**
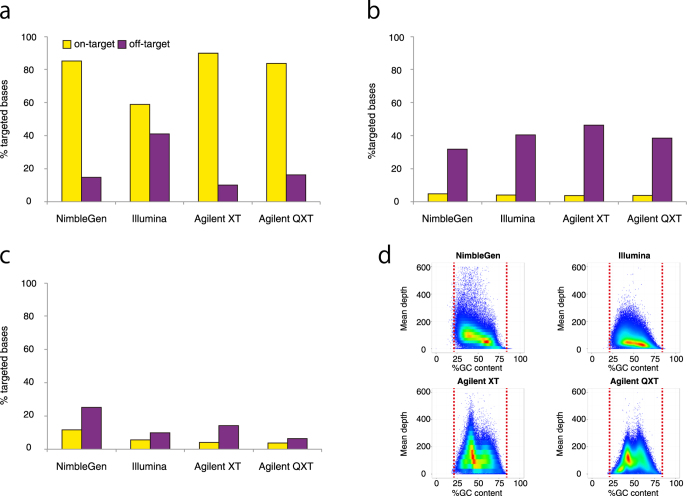
On-target enrichment and GC bias among platforms for NA18943. (**a**) On-target enrichment is represented by the percent of on-target (yellow) and off-target (purple) in each platform, when 75M reads were sequenced in NA18943. Of the sequenced bases, 14.8% were mapped to off-target regions with NimbleGen, 41.1% with Illumina, 10.1% with Agilent XT and 16.3% with Agilent QXT. The percent of regions that overlap RepeatMasker entries (**b**) and known segmental duplications (**c**) among on-target and off-target regions. (**d**) Density plot shows the correlation between mean read depth across target regions and GC content in each platform.

While the majority of sequence reads are mapped to on-target regions in each platform, some are mapped outside the on-target regions, called off-target regions. The percentage of reads mapped to the off-target regions is related strongly to the target enrichment efficiency. Therefore, we calculated the percentage of sequenced bases mapped to off-target regions and also investigated the regions they mapped to in the human genome. The Illumina platform showed a relatively low target enrichment efficiency compared to the other platforms, and required additional sequencing to capture a greater total number of bases in on-target regions (Fig. 3a for NA18943 and Supplementary Fig. S2a for NA18948). As expected, a greater proportion of sequenced bases in off-target regions were mapped to known segmental duplications and repeat regions identified by RepeatMasker (http://www.repeatmasker.org/) than those in on-target regions (Fig. 3b,c for NA18943 and Supplementary Fig. S2b and S2c for NA18948).

### GC content bias among platforms

We also investigated the relationship between mean sequencing depth in target regions and GC content for each platform. As expected, all platforms showed a bias against low GC content (<20%) and high GC content (>80%) regions. No major difference was observed between platforms. On the other hand, while the Illumina platform had relatively low read depths for on-target regions due to low target enrichment efficiency, it showed a more uniform distribution between 40% and 60% GC content in mean sequencing depth compared with the other platforms (Fig. 3d for NA18943 and Supplementary Fig. S2d for NA18948).

### Single nucleotide variation (SNV) detection

We performed SNV calling with our variant caller based on a multinomial probabilistic model, VCMM^18^. The total number of SNVs in on-target regions was 93,413, (NimbleGen), 69,372 (Illumina), 78,492 (Agilent XT) and 76,183 (Agilent QXT), when using the 75M read sets in the NA18943 sample. Of them, 21,759 SNVs (NimbleGen), 21,803 SNVs (Illumina), 21,930 SNVs (Agilent XT) and 21,246 SNVs (Agilent QXT) were detected in coding regions and 12,137 SNVs (NimbleGen), 6,124 SNVs (Illumina), 6,885 SNVs (Agilent XT) and 6,626 SNVs (Agilent QXT) in UTRs, and the remaining SNV were detected in intron and intergenic regions. While most of the SNVs in coding regions were detected in all platforms (19,143 SNVs), there were more NimbleGen specific UTR SNVs than UTR SNVs common to all platforms (NimbleGen-specific; 6,042 SNVs, common; 4,274 SNVs). This is the result of differences in the target regions among the platforms (Fig. 1b,c). The results were similar in the other sample, NA18948 (Supplementary Table S2).

Although Agilent XT and Agilent QXT have identical target regions, differences in SNV calls were observed. We therefore investigated the common variants and platform specific variants between the Agilent platforms. While most of the SNVs were commonly detected in both platforms (20,957 SNVs in coding regions, 6,228 SNVs in UTRs for NA18943), platform specific calls were observed in coding regions (XT specific: 973 SNVs, QXT specific: 289 SNVs) and in UTRs (XT specific: 657 SNVs, QXT specific: 398 SNVs). Most of these were in regions of low read depth (XT: 486/973 SNVs, QXT: 147/289 SNVs in coding regions, XT: 384/657, QXT: 337/398 in UTRs). Many of the remaining SNVs could be explained by mapping errors due to proximity to repeat regions or indels (XT: 87/487 SNVs, QXT: 50/142 SNVs in coding regions; XT: 41/273, QXT: 21/61 in UTRs). The remaining SNVs could be due to the different methods used for genomic DNA (gDNA) fragmentation. These platform specific SNVs were also distributed across all chromosomes, and no major difference was observed between chromosomes. Similar results were observed for the other sample, NA18948 (Supplementary Table S3).

We next evaluated the accuracy of the SNV calls for each platform by genotyping the same individuals on the Illumina HumanOmni2.5-Quad BeadChip. There was no difference in the concordance rate, 99.96%, across the platforms (Table 1 for NA18943 and Supplementary Table S4 for NA18948). We also estimated the ratio of heterozygous to homozygous SNVs in coding regions for each platform and found that Illumina and Agilent QXT detected more homozygous SNVs than the other two platforms (Supplementary Table S5). As this difference may be due to the method used for genomic DNA (gDNA) fragmentation, we counted the number of homozygous SNVs uniquely detected in each platform. In total, 332 unique homozygous SNVs were obtained across all four platforms. Of them, 260 (78.3%) were detected for NA18943 in the Illumina or Agilent QXT platform data, which enzymatically fragments the gDNA (256/328 = 78.1% for NA18948). A significant difference was observed when comparing the methods used for the gDNA fragmentation (p = 2.94e–14 for NA18943, p = 7.75e–14 for NA18948, two-tailed Fisher’s exact test). This result demonstrated that platforms which enzymatically fragment the gDNA detect more homozygous SNVs than those using sonicated gDNA.

### Short insertion and deletion (indel) detection

Short indel calling was also conducted by VCMM^18^. The number of indels detected in on-target regions was 16,795 (NimbleGen), 12,981 (Illumina), 11,935 (Agilent XT) and 12,270 (Agilent QXT), when using the 75M read sets in the NA18943 sample. Of them, 520 indels (NimbleGen), 642 indels (Illumina), 612 indels (Agilent XT) and 569 indels (Agilent QXT) were detected in coding regions, and 2,818 indels (NimbleGen), 1,021 indels (Illumina), 973 indels (Agilent XT) and 947 indels (Agilent QXT) in UTRs. As was the case with SNV detection, many of the indels detected in coding regions were shared among all platforms (shared; 366 indels), and many indels in the UTRs were NimbleGen platform specific (NimbleGen-specific; 1,999 indels, shared; 425 indels). Most of indels were commonly detected in both Agilent platforms (478 indels in coding regions, 756 indels in UTRs for NA18943), though some were platform specific to coding regions (XT specific: 134 indels, QXT specific: 91 indels) and UTRs (XT specific: 217 indels, QXT specific: 191 indels). Most of this disparity is due to the effect of low read depth (XT: 92/134 indels, QXT: 23/91 indels in coding regions, XT: 142/217, QXT: 91/191 in UTRs). Many of the remaining indels were located in or near repeat regions (XT: 16/42 indels, QXT: 38/68 indels in coding regions; XT: 28/75, QXT: 44/100 in UTRs). The remaining small number of indels could be due to the different methods used for genomic DNA (gDNA) fragmentation. Similar results were also found in the other sample, NA18948 (Supplementary Table S2 and Supplementary Table S3). While we also counted the number of homozygous indels uniquely detected in each platform, no significant difference was observed in indel detection when comparing the methods used for the gDNA fragmentation.

We also investigated the distribution of indel length in coding regions and found that all platforms showed a similar distribution, ranged from −39 to +42 bases in size. More than 92% of the indels detected were less than ten bases long for all platforms. The lengths of approximately half of the indels were multiples of three, reflecting a strong selective pressure against deleterious frameshift mutations in coding regions.

### Coverage of medically interesting rare mutations

For WXS analysis, the identification of disease-causing rare mutations is of particular importance. We therefore investigated the coverage of medically interesting rare mutations, present in the Human Gene Mutation Database (HGMD, Professional 2014.2) but absent from public databases (1000 Genomes Project^19^ and NHLBI Exome Variant Server^20^ [accessed June 2012]). The number of the mutations was 71,256. Of them, more than 97.8% had at least ten times coverage in Illumina: 96.7% in Agilent XT, 96.1% in Agilent QXT, and 95.1% in NimbleGen (Supplementary Table S6). The Illumina platform showed the highest performance in the coverage in both samples (Supplementary Table S6). We also investigated why Illumina achieved the highest sequence coverage in the medically interesting rare mutations, despite the fact that Agilent XT achieved the highest sequence coverage in the coding regions. This resulted from the difference of target region design between platforms and the uniform distribution of GC content in mean sequencing depth. In fact, only 432 of the 71,256 mutations were in non-target regions for Illumina, whereas 1,117 were for Agilent XT. In addition, Agilent XT only covered 9 of the 432 mutations not targeted by Illumina, whereas Illumina covered 631 of the 1,117 mutations not targeted by Agilent XT for NA18943 (11/432 and 623/1,117 for NA18948). However, it should be noted that this result demonstrates the high power of the all platforms for the coverage of medically interesting rare mutations.

## Discussion

The performance comparison analysis of current exome enrichment platforms will be helpful to investigators when selecting the best platform for their research^13^,^14^,^15^,^16^,^17^. Therefore, we comprehensively evaluated the latest version of the four major exome platforms from three manufactures with respect to four parameters: target enrichment efficiency, GC bias, sensitivity in SNV and short indel detection and coverage of medically interesting mutations. Basically, all platforms showed a high performance in target enrichment efficiency and covered large percentage of the coding regions. Most of the coding regions, as well as medically interesting mutations, had at least ten times coverage when 75M reads were sequenced.

On the other hand, most of investigators of exome sequencing, especially in the medical sciences, are interested in the small difference in coverage of coding regions, because the difference directly reflects the ability to identify rare variants in the coding regions. In this case, Agilent XT and Illumina platforms show higher coverage than the other two platforms for coding regions. A similar tendency is also seen in the number of SNVs and short indels identified as well as medically interesting rare mutations having at least ten times coverage.

For comparison of GC bias, all four platforms displayed a negative correlation between sequencing coverage and extreme high or small GC contents as expected. No major difference is observed between the four platforms. However, less GC bias is observed between 40% and 60% in the Illumina platform compared to the other three. This difference may contribute to the Illumina platform’s high coverage of coding regions despite its target enrichment efficiency being lower than the other three.

Although the Illumina platform had a higher percentage of off-target mapped bases than the other three, the percentage of bases mapped to repeat regions, including those in the off-target regions, was similar to the others. Therefore, the Illumina platform produced much more sequence that mapped to the off-target regions, but not to repeat regions, suggesting that some of these sequenced bases may have a potential use in subsequent exome sequencing analysis. In addition, the Illumina platform showed the highest performance for the coverage of medically interesting rare mutations, even though the Agilent XT platform achieved the highest sequence coverage in the coding regions. It would appear this is due to the difference in target region design between platforms and the uniform distribution of GC content in mean sequencing depth.

A difference in the ratio of heterozygous to homozygous SNVs was also observed between platforms, and Illumina and Agilent QXT detected more homozygous SNVs than the other platforms. Since these two platforms enzymatically fragment gDNA, sequence selection bias of transposons might have an impact on the difference in the number of homozygous SNVs detected. In other words, these platforms using sonicated gDNA might be inadequate for the analysis that detects somatic mutations from data with low heterozygous frequency.

Owing to rapid evolution and improvement of exome capture platforms, the difference in performance among platforms is becoming smaller. On the other hand, the sequencing cost varies greatly between platforms. The Illumina platform is the cheapest among the four platforms tested. However, the Illumina platform also shows the lowest target enrichment efficiency. Therefore, when selecting most suitable platform, investigators will need to consider the cost of reagents to reach the required coverage. Other variables of interest are ease of library construction and the amount of input DNA required. Library construction is easiest for the Illumina and Agilent QXT platforms, and the amount of input DNA required is tiny for the Illumina platform, as compared to the other platforms. These differences should be also taken into account when selecting the appropriate platform.

## Materials and Methods

### DNA Sample

DNA samples of HapMap-JPT NA18943 and NA18948 were obtained from Coriell, where lymphoblastoid cell lines were established by Epstein-Barr virus (Human herpesvirus 4)-mediated transformation of peripheral blood mononuclear cells. NA18943 and NA18948 satisfied the following criteria: genotyped in the International HapMap project, male sample, and without a karyotype abnormality report.

### Exome capture library and whole-exome sequencing

We sequenced libraries generated from genomic DNA derived from peripheral blood mononuclear cells of Japanese descent. Each exome captured sequencing library was produced from one of four different technologies: Roche/NimbleGen’s SeqCap EZ Human Exome Library v3.0, Illumina’s Nextera Rapid Capture Exome (v1.2), Agilent’s SureSelect XT Human All Exon v5 and Agilent’s SureSelect QXT. Each exome capture platform method was performed according to the manufacturer’s instructions except for Illumina platform. For the Illumina platform, we used 100 ng of total input genomic DNA. The captured DNA was sequenced using the Illumina HiSeq2500 platform with paired-end reads of 161bp for insert libraries according to the manufacturer’s instructions. We deposited all DNA sequence data used in this study to the National Bioscience Database Center (NBDC) Human Database (http://humandbs.biosciencedbc.jp/).

### Exome sequence data analysis

Read sequences were mapped using the Burrows-Wheeler Aligner (BWA: version 0.6.1)^21^ to the human reference genome (GRCh37). Duplicate PCR reads were identified and removed using SAMtools (version 0.1.8)^22^ and in-house software. After filtering by pair mapping distance, mapping uniqueness and pair orientation, the mapping result files were converted into pileup format using SAMtools. Variant calling was conducted based on methods we have published elsewhere, VCMM^18^. We used the following quality control filters: (i) alignments near putative indels were refined using GATK^23^, and (ii) a stand bias filter excluded variants whose alternative allele was preferentially found in one of the two available read orientations at the site.

### Accuracy evaluation using SNP typing platforms

In order to evaluate accuracy of variant calls in each platform, we compared our SNV calls results with the concordant genotypes from a SNP genotyping platform: Illumina HumanOmni2.5-Quad BeadChip. Of the 208,228 SNPs, 203,829 with NimbleGen, 204,033 with Illumina, 207,381 with Agilent XT and 206,777 with Agilent QXT for NA18943, and 203,884 with NimbleGen, 203,165 with Illumina and 207,209 with Agilent XT and 206,745 with Agilent QXT for NA18948 were eventually used for the estimation of the concordance rate.

## Additional Information

**How to cite this article**: Shigemizu, D. *et al.* Performance comparison of four commercial human whole-exome capture platforms. *Sci. Rep.* **5**, 12742; doi: 10.1038/srep12742 (2015).

## Supplementary Material

Supplementary Information

## Figures and Tables

**Table 1:** Estimation of accuracy of SNVs using SNP genotyping platform for NA18943.

	**Genotyping array**†	**WXS**†	**NimbleGen**	**Illumina**	**Agilent XT**	**Agilent QXT**
Not analyzed			4,544	4,360	847	1,451
Concordance (a) (a/a+b)			203,754 (99.96%)	203,947 (99.96%)	207,294 (99.96%)	206,702 (99.96%)
Discordance	Ho	Ht	20	12	25	16
	Ho	Ho*	34	36	36	33
	Ht	Ht*	1	2	2	2
	Ht	Ho	20	36	24	24
	Total (b)		75	86	87	75

^†^Ht; Heterozygous genotype, Ho; Homozygous genotype.

^*^Different genotype to that of genotyping array.

## References

[b1] Lalonde E. *et al.* Unexpected allelic heterogeneity and spectrum of mutations in Fowler syndrome revealed by next-generation exome sequencing. Hum Mutat 31, 918–23 (2010). 20518025 10.1002/humu.21293

[b2] Ng S.B. *et al.* Exome sequencing identifies the cause of a mendelian disorder. Nat Genet 42, 30–5 (2010). 19915526 10.1038/ng.499 PMC2847889

[b3] Rusk N. & Kiermer V. Primer: Sequencing–the next generation. Nat Methods 5, 15 (2008). 18175411 10.1038/nmeth1155

[b4] Mardis E.R. Next-generation DNA sequencing methods. Annu Rev Genomics Hum Genet 9, 387–402 (2008). 18576944 10.1146/annurev.genom.9.081307.164359

[b5] Metzker M.L. Sequencing technologies - the next generation. Nat Rev Genet 11, 31–46 (2010). 19997069 10.1038/nrg2626

[b6] Chang H. *et al.* Exome sequencing reveals comprehensive genomic alterations across eight cancer cell lines. PLoS One 6, e21097 (2011). 21701589 10.1371/journal.pone.0021097 PMC3118809

[b7] Challis D. *et al.* An integrative variant analysis suite for whole exome next-generation sequencing data. BMC Bioinformatics 13, 8 (2012). 22239737 10.1186/1471-2105-13-8 PMC3292476

[b8] Kiezun A. *et al.* Exome sequencing and the genetic basis of complex traits. Nat Genet 44, 623–30 (2012). 22641211 10.1038/ng.2303 PMC3727622

[b9] Choi M. *et al.* Genetic diagnosis by whole exome capture and massively parallel DNA sequencing. Proc Natl Acad Sci U S A 106, 19096–101 (2009). 19861545 10.1073/pnas.0910672106 PMC2768590

[b10] Wei X. *et al.* Exome sequencing identifies GRIN2A as frequently mutated in melanoma. Nat Genet 43, 442–6 (2011). 21499247 10.1038/ng.810 PMC3161250

[b11] Varela I. *et al.* Exome sequencing identifies frequent mutation of the SWI/SNF complex gene PBRM1 in renal carcinoma. Nature 469, 539–42 (2011). 21248752 10.1038/nature09639 PMC3030920

[b12] Agrawal N. *et al.* Exome sequencing of head and neck squamous cell carcinoma reveals inactivating mutations in NOTCH1. Science 333, 1154–7 (2011). 21798897 10.1126/science.1206923 PMC3162986

[b13] Asan *et al.* Comprehensive comparison of three commercial human whole-exome capture platforms. Genome Biol 12, R95 (2011). 21955857 10.1186/gb-2011-12-9-r95 PMC3308058

[b14] Clark M.J. *et al.* Performance comparison of exome DNA sequencing technologies. Nat Biotechnol 29, 908–14 (2011). 21947028 10.1038/nbt.1975 PMC4127531

[b15] Parla J.S. *et al.* A comparative analysis of exome capture. Genome Biol 12, R97 (2011). 21958622 10.1186/gb-2011-12-9-r97 PMC3308060

[b16] Sulonen A.M. *et al.* Comparison of solution-based exome capture methods for next generation sequencing. Genome Biol 12, R94 (2011). 21955854 10.1186/gb-2011-12-9-r94 PMC3308057

[b17] Chilamakuri C.S. *et al.* Performance comparison of four exome capture systems for deep sequencing. BMC Genomics 15, 449 (2014). 24912484 10.1186/1471-2164-15-449 PMC4092227

[b18] Shigemizu D. *et al.* A practical method to detect SNVs and indels from whole genome and exome sequencing data. Sci Rep 3, 2161 (2013). 23831772 10.1038/srep02161 PMC3703611

[b19] Clarke L. *et al.* The 1000 Genomes Project: data management and community access. Nat Methods 9, 459–62 (2012). 22543379 10.1038/nmeth.1974 PMC3340611

[b20] Fu W. *et al.* Analysis of 6,515 exomes reveals the recent origin of most human protein-coding variants. Nature 493, 216–20 (2013). 23201682 10.1038/nature11690 PMC3676746

[b21] Li H. & Durbin R. Fast and accurate short read alignment with Burrows-Wheeler transform. Bioinformatics 25, 1754–60 (2009). 19451168 10.1093/bioinformatics/btp324 PMC2705234

[b22] Li H. *et al.* The Sequence Alignment/Map format and SAMtools. Bioinformatics 25, 2078–9 (2009). 19505943 10.1093/bioinformatics/btp352 PMC2723002

[b23] McKenna A. *et al.* The Genome Analysis Toolkit: a MapReduce framework for analyzing next-generation DNA sequencing data. Genome Res 20, 1297–303 (2010). 20644199 10.1101/gr.107524.110 PMC2928508

