# Supplementary material for: Performance comparison of four commercial human whole-exome capture platforms
Source: Sci Rep. 2015 Aug 3;5:12742. doi: 10.1038/srep12742 (PMC4522667; doi:10.1038/srep12742)
Supplement: Supplementary Information [file srep12742-s1.doc]

**Supplementary Information**

**Performance comparison of four commercial human whole-exome capture platforms**

Daichi Shigemizu^1^, Yukihide Momozawa^2^, Testuo Abe^1^, Takashi Morizono^1^, Keith A Boroevich^1^, Sadaaki Takata^2^, Kyota Ashikawa^2^, Michiaki Kubo^2*^ and Tatsuhiko Tsunoda^1*^

^1^ Laboratory for Medical Science Mathematics, RIKEN Center for Integrative Medical Sciences, Yokohama, Japan.

^2^ Laboratory for Genotyping Development, RIKEN Center for Integrative Medical Sciences, Yokohama, Japan.

^*^These authors contributed equally to this project and should be considered co-corresponding authors.

*Correspondence should be addressed to T.Tsunoda (tatsuhiko.tsunoda@riken.jp)

**Supplementary Table S1 | Mapping rate and PCR duplication rate for each platform.**

| Sample | Platform | Reads sequenced (a) | Reads mapped (b) | Mapping rate (b/a) | Uniquely mapped (c) | % PCR duplication rates (c/a) |
| --- | --- | --- | --- | --- | --- | --- |
| NA18943 | NimbleGen | 75,000,000 | 73,302,020 | 97.74 | 70,901,674 | 3.20 |
|  | Illumina | 75,000,000 | 72,629,360 | 96.84 | 65,708,849 | 9.23 |
|  | Agilent XT | 75,000,000 | 73,887,070 | 98.52 | 65,784,688 | 10.80 |
|  | Agilent QXT | 75,000,000 | 74,190,030 | 98.92 | 63,034,534 | 14.87 |
| NA18948 | NimbleGen | 75,000,000 | 73,423,042 | 97.90 | 71,038,922 | 3.18 |
|  | Illumina | 75,000,000 | 72,308,906 | 96.41 | 65,679,539 | 8.84 |
|  | Agilent XT | 75,000,000 | 73,053,962 | 97.41 | 62,961,126 | 13.46 |
|  | Agilent QXT | 75,000,000 | 74,152,142 | 98.87 | 63,057,969 | 14.79 |

**Supplementary Table S2 | SNV and short indel detection on-target regions for each platform.**

|  | Sample | Platform | Total | Coding regions (shared) | UTRs (shared) |
| --- | --- | --- | --- | --- | --- |
| SNV | NA18943 | NimbleGen | 93,413 | 21,759 (19,143) | 12,137 (4,274) |
|  |  | Illumina | 69,372 | 21,803 (19,143) | 6,124 (4,274) |
|  |  | Agilent XT | 78,492 | 21,930 (19,143) | 6,885 (4,274) |
|  |  | Agilent QXT | 76,183 | 21,246 (19,143) | 6,626 (4,274) |
|  | NA18948 | NimbleGen | 93,125 | 21,622 (18,972) | 12,059 (4,211) |
|  |  | Illumina | 68,084 | 21,505 (18,972) | 6,017 (4,211) |
|  |  | Agilent XT | 80,885 | 22,187 (18,972) | 7,133 (4,211) |
|  |  | Agilent QXT | 76,162 | 20,983 (18,972) | 6,603 (4,211) |
| Indel | NA18943 | NimbleGen | 16,795 | 520 (366) | 2,818 (425) |
|  |  | Illumina | 12,981 | 642 (366) | 1,021 (425) |
|  |  | Agilent XT | 11,935 | 612 (366) | 973 (425) |
|  |  | Agilent QXT | 12,270 | 569 (366) | 947 (425) |
|  | NA18948 | NimbleGen | 16,898 | 516 (363) | 2,877 (428) |
|  |  | Illumina | 12,917 | 637 (363) | 1,012 (428) |
|  |  | Agilent XT | 11,810 | 598 (363) | 978 (428) |
|  |  | Agilent QXT | 12,301 | 539 (363) | 946 (428) |

**Supplementary Table S3 | common and platform specific SNV and short indel detection on-target regions bewteen two Agilent platforms.**

|  |  |  | SNV |  |  |  | Indel |  |  |  |
| --- | --- | --- | --- | --- | --- | --- | --- | --- | --- | --- |
|  |  |  | XT |  | QXT |  | XT |  | QXT |  |
|  |  |  | # | % | # | % | # | % | # | % |
| NA18943 | CDS | Common | 20,957 | 95.6 | 20,957 | 98.6 | 478 | 78.1 | 478 | 84.0 |
|  |  | Specific | 973 | 4.4 | 289 | 1.4 | 134 | 21.9 | 91 | 16.0 |
|  |  | Low depth | 486 | 2.2 | 147 | 0.7 | 92 | 15.0 | 23 | 4.0 |
|  |  | Repeat/Indel | 87 | 0.4 | 50 | 0.2 | 16 | 2.6 | 38 | 6.7 |
|  |  | Others | 400 | 1.8 | 92 | 0.4 | 26 | 4.2 | 30 | 5.3 |
|  | UTR | Common | 6,228 | 90.5 | 6,228 | 94.0 | 756 | 77.7 | 756 | 79.8 |
|  |  | Specific | 657 | 9.5 | 398 | 6.0 | 217 | 22.3 | 191 | 20.2 |
|  |  | Low depth | 384 | 5.6 | 337 | 5.1 | 142 | 14.6 | 91 | 9.6 |
|  |  | Repeat/indel | 41 | 0.6 | 21 | 0.3 | 28 | 2.9 | 44 | 4.6 |
|  |  | Others | 232 | 3.4 | 40 | 0.6 | 47 | 4.8 | 56 | 5.9 |
| NA18948 | CDS | Common | 20,733 | 93.4 | 20,733 | 98.8 | 451 | 75.4 | 451 | 83.7 |
|  |  | Specific | 1,454 | 6.6 | 250 | 1.2 | 147 | 24.6 | 88 | 16.3 |
|  |  | Low depth | 552 | 2.5 | 129 | 0.6 | 97 | 16.2 | 29 | 5.4 |
|  |  | Repeat/Indel | 101 | 0.5 | 35 | 0.2 | 21 | 3.5 | 28 | 5.2 |
|  |  | Others | 801 | 3.6 | 86 | 0.4 | 29 | 4.8 | 31 | 5.8 |
|  | UTR | Common | 6,163 | 86.4 | 6,163 | 93.3 | 765 | 78.2 | 765 | 80.9 |
|  |  | Specific | 970 | 13.6 | 440 | 6.7 | 213 | 21.8 | 181 | 19.1 |
|  |  | Low depth | 463 | 6.5 | 364 | 5.5 | 140 | 14.3 | 94 | 9.9 |
|  |  | Repeat/indel | 61 | 0.9 | 29 | 0.4 | 25 | 2.6 | 44 | 4.7 |
|  |  | Others | 446 | 6.3 | 47 | 0.7 | 48 | 4.9 | 43 | 4.5 |

**Supplementary Table S4 | Estimation of accuracy of SNVs using SNP genotyping platform for NA18948.**

|  | Genotyping array† | WXS† | NimbleGen | Illumina | Agilent XT | Agilent QXT |
| --- | --- | --- | --- | --- | --- | --- |
| Not analyzed |  |  | 4,489 | 5,228 | 1,019 | 1,483 |
| Concordance (a) (a / a+b) |  |  | 203,798  (99.96%) | 203,080  (99.96%) | 207,120  (99.96%) | 206,666  (99.96%) |
| Discordance | Ho | Ht | 18 | 12 | 26 | 16 |
|  | Ho | Ho* | 37 | 38 | 38 | 36 |
|  | Ht | Ht* | 5 | 5 | 5 | 5 |
|  | Ht | Ho | 26 | 30 | 20 | 22 |
|  | Total (b) |  | 86 | 85 | 89 | 79 |

†: Ht; Heterozygous genotype, Ho; Homozygous genotype.

*: Different genotype to that of genotyping array

**Supplementary Table S5 | Ratio of heterozygous to homozygous SNVs in coding regions for each platform.**

| Sample | Platform | Total | Heterozygous | Homozygous |
| --- | --- | --- | --- | --- |
| NA18943 | NimbleGen | 21,759 | 13,018 (59.8%) | 8,741 (40.2%) |
|  | Illumina | 21,803 | 12,911 (59.2%) | 8,892 (40.8%) |
|  | Agilent XT | 21,930 | 13,003 (59.3%) | 8,927 (40.7%) |
|  | Agilent QXT | 21,246 | 12,469 (58.7%) | 8,777 (41.3%) |
| NA18948 | NimbleGen | 21,622 | 12,943 (59.9%) | 8,679 (40.1%) |
|  | Illumina | 21,505 | 12,722 (59.2%) | 8,783 (40.8%) |
|  | Agilent XT | 22,187 | 13,311 (60.0%) | 8,876 (40.0%) |
|  | Agilent QXT | 20,983 | 12,259 (58.4%) | 8,724 (41.6%) |

**Supplementary Table S6 | Coverage of medically interesting rare mutations.**

| Sample | Platform | ≥1x | %≥1x | ≥10x | %≥10x |
| --- | --- | --- | --- | --- | --- |
| NA18943 | NimbleGen | 69,820 | 97.98 | 67,948 | 95.36 |
|  | Illumina | 70,455 | 98.88 | 69,745 | 97.88 |
|  | Agilent XT | 69,744 | 97.88 | 68,972 | 96.79 |
|  | Agilent QXT | 69,704 | 97.82 | 68,561 | 96.22 |
| NA18948 | NimbleGen | 69,783 | 97.93 | 67,819 | 95.18 |
|  | Illumina | 70,458 | 98.88 | 69,701 | 97.82 |
|  | Agilent XT | 69,708 | 97.83 | 68,905 | 96.70 |
|  | Agilent QXT | 69,686 | 97.80 | 68,484 | 96.11 |

**Supplementary Figure S1 | Coverage of target regions for each platform for NA18948.** (a) The percent of total targeted bases covered with more than or equal to specified depths in the NA18948 sample. For target regions, 88.5% had at least ten times sequence coverage with NimbleGen, 88.2% with Illumina, 90.7% with Agilent XT, and 92.1% with Agilent QXT. The percent of on-target regions (b) and coding regions (c) covered with at least 10-fold read depth at increasing read counts. When 75M reads were sequenced, 94.0% of coding regions had at least ten times coverage on average with NimbleGen, 95.5% with Illumina, 95.9% with Agilent XT and 95.3% with Agilent QXT.

**Supplementary Figure S2 | On-target enrichment and GC bias among platforms for NA18948.** (a) On-target enrichment is represented by the percent of on-target (yellow) and off-target (purple) in each platform, when 75M reads were sequenced in the NA18948 sample. Of sequenced bases, 14.7% were mapped to off-target regions with NimbleGen, 39.8% with Illumina, 11.9% with Agilent XT, and 17.2% with Agilent QXT. The percent of regions that overlap RepeatMasker entries (b) and known segmental duplications (c) among on-target and off-target regions. (d) Density plot shows the correlation between mean read depth across target regions and GC content in each platform.
